# Reduced social participation among seniors with self-reported visual impairment and glaucoma

**DOI:** 10.1371/journal.pone.0218540

**Published:** 2019-07-23

**Authors:** Shicheng Jin, Graham E. Trope, Yvonne M. Buys, Elizabeth M. Badley, Kednapa Thavorn, Peng Yan, Harrish Nithianandan, Ya-Ping Jin

**Affiliations:** 1 Department of Ophthalmology and Vision Sciences, University of Toronto, Toronto, Canada; 2 Division of Health Care and Outcomes Research, Toronto Western Research Institute, University Health Network, Toronto, Canada; 3 Dalla Lana School of Public Health, University of Toronto, Toronto, Canada; 4 Clinical Epidemiology Program, Ottawa Hospital Research Institute, The Ottawa Hospital, Ottawa, Ontario, Canada, School of Epidemiology and Public Health, University of Ottawa, Ottawa, Canada; 5 Faculty of Medicine, University of Ottawa, Ottawa, Canada; 6 Institute of Medical Science, University of Toronto, Toronto, Canada; Indiana University Purdue University at Indianapolis, UNITED STATES

## Abstract

**Objective:**

Social participation benefits health. We assessed the relationship between self-reported visual impairment (VI) and glaucoma versus seniors’ social participation.

**Methods:**

Data from individuals aged ≥65 years responding to the Canadian Community Health Survey Healthy Aging 2008/2009 (n = 16,369) was analyzed. Participation in eight social activities by seniors with and without self-reported VI or glaucoma was compared.

**Results:**

Seniors with VI had significantly reduced participation (p<0.05) in sports/physical activities (18.0% vs. 33.6%), family/friendship activities outside the household (39.7% vs. 53.0%), service club/fraternal organization activities (11.4% vs. 18.4%), volunteer/charity work (13.4% vs. 24.9%), educational/cultural activities (16.2% vs. 24.5%), and other social recreational activities (21.6% vs. 31.0%) compared to those without VI. Differences in participation in church/religious activities (40.6% vs. 44.5%) and community/professional association activities (15.3% vs. 18.0%) were non-significant between seniors with and without VI. Seniors with glaucoma versus those without had significantly reduced participation (p<0.05) in family/friendship activities (46.6% vs. 52.9%), sports/physical activities (26.0% vs. 33.6%) and volunteer/charity work (20.4% vs. 24.9%). No participation in any social activity was significantly higher among seniors with VI versus those without (10.1% vs. 2.9%, p<0.05), but was similar among seniors with and without glaucoma (3.9% vs. 3.1%, p>0.05). After adjusting for the effects of age, sex, education, household income, ethnicity, job status and chronic diseases (adjusted odds ratio, aOR = 3.4 (95% confidence interval (CI) 2.0–5.8), seniors with VI but no glaucoma were more likely not to engage in any social activities compared to seniors without VI and no glaucoma. Seniors with glaucoma but without VI had a similar level of non-participation (aOR = 0.9, 95%% CI 0.5–1.7).

**Conclusions:**

Significantly reduced social participation was found across six community activities among seniors with self-reported VI and in three activities among those with self-reported glaucoma. Policies and programs that help seniors with VI or glaucoma engage in social activities are needed.

## Introduction

Globally, loss of central vision, or visual impairment (VI), impacts 70 million people over the age of 70.[1] The top causes of VI include uncorrected refractive error, cataract, age-related macular degeneration, and diabetic retinopathy.[2] Glaucoma, a leading cause of irreversible blindness worldwide, is a complex disease in which damage to the optic nerve leads to progressive and permanent vision loss.[3] A common feature of glaucoma is the initial loss of peripheral vision followed by central vision in severe cases [3] The prevalence of VI and the prevalence of glaucoma increases significantly with increasing age, and is particularly higher among individuals aged 65 years or older.[4–8]

In 2016, the WHO endorsed the *Global Strategy and Action Plan on Ageing and Health* introducing the concept of “healthy ageing” as the focal point for governments to develop policies to meet the needs of the aging population.[9,10] Social participation is recognized as a key component for “healthy ageing”.[9,10] This is because participation in social and community activities has been shown to lower the risk of all-cause mortality[11–15], motor decline[16], cognitive decline[17], depressive symptoms[18,19], and psychological distresses.[20] Since 2001, “participation” has been proposed by WHO as one of four elements in the biopsychosocial model for disease, namely impairment, activity limitation, participation and environment.[21]

In the literature, many studies on vision disorders have focused on the link between the element “impairment” (e.g. glaucoma) and the element “activity limitation” (e.g. limitation in driving activity). For instance, reports have shown that VI (i.e. the “impairment” element in the WHO biopsychosocial disease model) is associated with significantly decreased activities of reading and mobility[22–25] and that the ‘impairment’ from glaucoma was associated with ‘activity limitation’ such as driving, mobility, and reading.[26–32] However, fewer studies have examined the link between the element ‘impairment’ and the element of ‘participation’, defined as ‘involvement in life situations’ by the WHO.[21] Of the available studies on participation among seniors with VI, limitations include small sample size (n = 173)[33], recruiting patients from a clinic setting[33], having a broad definition of social participation[33–37] or non-recent (1994) data.[38]

Given our rapidly growing aging population, the number of seniors affected by vision disorders will increase in the coming years.[39,40] Understanding the association between VI and glaucoma versus social participation among seniors will inform policies and programs to help seniors with vision disorders optimize their quality of life while aging. Using data collected externally by Statistics Canada from a nationwide randomly selected sample, we aim to examine the relationship between VI and glaucoma versus seniors’ participation across a wide range of social activities.

## Methods

### Data collection

Population-based survey data from the Canadian Community Health Survey—Healthy Aging (CCHS-HA) 2008–2009 was analyzed. The CCHS-HA was a health survey run by Statistics Canada, a government of Canada agency commissioned with collecting population-level data.[41] Individuals aged 45 years or older living in private dwellings in the 10 Canadian provinces (n = 30,865) were randomly selected.[41] Those living in long-term care institutions, full-time members of the Canadian Forces and residents of certain remote regions were excluded from the survey.[41] The overall survey response rate was 74.4%.[41] The CCHS-HA contains the largest, most recently available data regarding VI, glaucoma and social participation in Canada. Details regarding CCHS-HA’s survey are available in a published report.[41] During the process of data collection, data access and analysis, Statistics Canada took strict measures to protect respondent’s informed and voluntary consent right and confidentiality.[42] Ethics approval for this study was granted by the Research Ethics Board at the University of Toronto.

Data on self-reported VI was obtained by asking:

- Are you usually able to see well enough to read ordinary newsprint *without* glasses or contact lenses?
- Are you usually able to see well enough to read ordinary newsprint *with* glasses or contact lenses?
- Are you able to see at all?
- Are you able to see well enough to recognize a friend on the other side of the street *without* glasses or contact lenses?
- Are you able to see well enough to recognize a friend on the other side of the street *with* glasses or contact lenses?

Answers to the above questions were grouped into 5 mutually exclusive groups by Statistics Canada:

1. No visual problems;
2. Problems corrected by lenses (distance, close or both);
3. Problems seeing distance with or without correction;
4. Problems seeing close with or without correction; and
5. Problems seeing close and distance, or no sight at all.

In this study, we defined self-reported VI as impairment uncorrected by lenses for distance vision (group 3), near vison (group 4) or both, or no sight at all (group 5).

Respondents were classified to have glaucoma if they self-reported having glaucoma that had lasted or was expected to last six months or more and that had been diagnosed by a health professional.[43]

Social participation was assessed by asking how often in the past 12 months respondents had participated in eight areas of activity.[43] The eight areas of activity were: 1) family or friendship activities outside the household; 2) church or religious activities such as services, committees or choirs; 3) sports or physical activities that involve other people; 4) educational and cultural activities involving other people such as attending courses, concerts or visiting museums; 5) service club or fraternal organization activities; 6) neighbourhood, community or professional association activities; 7) volunteer or charity work; and 8) any other recreational activities involving other people, including hobbies, bingo and other games. For each activity response options were: ‘at least once a day’, ‘at least once a week’, ‘at least once a month’, ‘at least once a year’ or ‘never’.

During analyses, answers of ‘at least once a day’, ‘at least once a week’, or ‘at least once a month’ were grouped together as ‘participation’ to avoid sparse data. Answers of ‘at least once a year’ or ‘never’ were considered as ‘non-participation’. However, for participation in ‘family or friendship activities outside the household’, ‘participation’ was defined differently, where answers of ‘at least once a day’ or ‘at least once a week’ were deemed as ‘participation’ due to the common occurrence of this activity.

Data on age, sex, ethnic background (Caucasians vs. non-Caucasians), highest level of education (without vs. with post-secondary degree), total household income (under middle level vs. middle level or higher), current job status (part-time or full-time workers vs. non-workers) and chronic conditions (having at least one of 25 chronic conditions surveyed vs. none) other than VI and glaucoma was self-reported.[43] Respondents aged 65 years or older were included in the main analyses for three reasons. First, seniors are the portion of the population most affected by VI and glaucoma as reported. This was confirmed in this study: the prevalence of self-reported VI was 1.0% in those aged 45–64 versus 3.8% (p<0.05) in those aged 65+. For glaucoma, the prevalence was 1.6% in the 45–64 year group versus 7.2% (p<0.05) in the 65+. Secondly, labour force participation rate is significantly different between people aged 45–64 and those aged 65+. Thirdly, people in the 65+ age group have finished raising children and are mostly retired. As a result, non-participation in social activities due to responsibilities of attending job and taking care of children may be different between individuals aged 65 years or older and those aged 45–64. However, to facilitate comparisons with prior studies, we also included the analyses in people aged 45+ and compared the results from people aged 65+ yrs versus 45+ yrs.

### Statistical analysis

We analyzed the raw de-identified data housed at the Research Data Centre (RDC) at the University of Toronto where participant’s confidentiality was strictly protected. Survey weights provided by Statistics Canada were used in all analyses. These weights accounted for the complex survey design and sample selections, adjustments for nonresponse, seasonal effects, and poststratification.[41] Weighted data are therefore more representative of the survey population and are required by Statistics Canada for reporting when producing population estimates.[41] The 95% confidence interval (CI) was constructed using bootstrap weights provided by Statistics Canada. Potential confounders (age, sex, highest level of education, total household income, ethnic background, current job status and chronic conditions other than self-reported VI and glaucoma) were adjusted for in a multiple logistic regression model. Since VI defined in this study mainly affects the central vision and loss of peripheral vision is featured in glaucoma and that both VI and glaucoma reduced senior’s participation in certain areas of social activities, we therefore chose seniors with no VI and no glaucoma as a reference group in the regression analysis.

## Results

In Canada, an estimated 163,100 (3.8%) seniors had self-reported VI, 312,000 (7.2%) had self-reported glaucoma and 30,900 (0.7%) had both self-reported VI and glaucoma in 2008/2009. The characteristics of respondents are shown in Table 1.

**Table 1:** Characteristics of respondents aged 65+ to the Canadian Community Health Survey–Healthy Aging (CCHS-HA) 2008–2009 (numbers reported are weighted, except for n–unweighted sample size).

	VI+*			VI-*		
	Glaucoma+	Glaucoma-	Total	Glaucoma+	Glaucoma-	Total
n (unweighted)	145	689	834	1,140	14,187	15,327
Mean age (years)	81.3	79.9	80.1^a^	77.3^b^	74.1^b^	74.3^a^
Female (%)	65.6	58.8	60.1	55.3	54.5	54.5
No post-secondary education (%)	63.4	63.1	63.2^a^	60.5	56.1	56.4^a^
Caucasians (%)	80.0	89.0	87.2	89.9	91.7	91.5
Household income distribution less then middle level (%)**	77.9	71.9	73.2^a^	62.3	61.1	61.1^a^
Work full-time or part-time (%)	2.6	4.4	4.0^a^	7.6^b^	13.4^b^	13.0^a^
Has ≥1 chronic health condition(s) diagnosed by a health professional (%)***	98.3	95.5	96.0^a^	95.3^b^	90.6^b^	90.9^a^

*: VI: Visual impairment; +: presence of the condition; -: absence of the condition

**: Respondent’s household income distribution is less than the fifth decile of a total of ten deciles, which was calculated based on the adjusted ratio of respondent’s total household income to the low income cut-off corresponding to their household and community size***: not including VI and glaucoma.

a: p<0.05 for VI+ (total) vs VI- (total)

b: p<0.05 for VI-Glaucoma+ vs VI-Glaucoma-.

### Social participation among people with and without visual impairment

**Fig 1:**
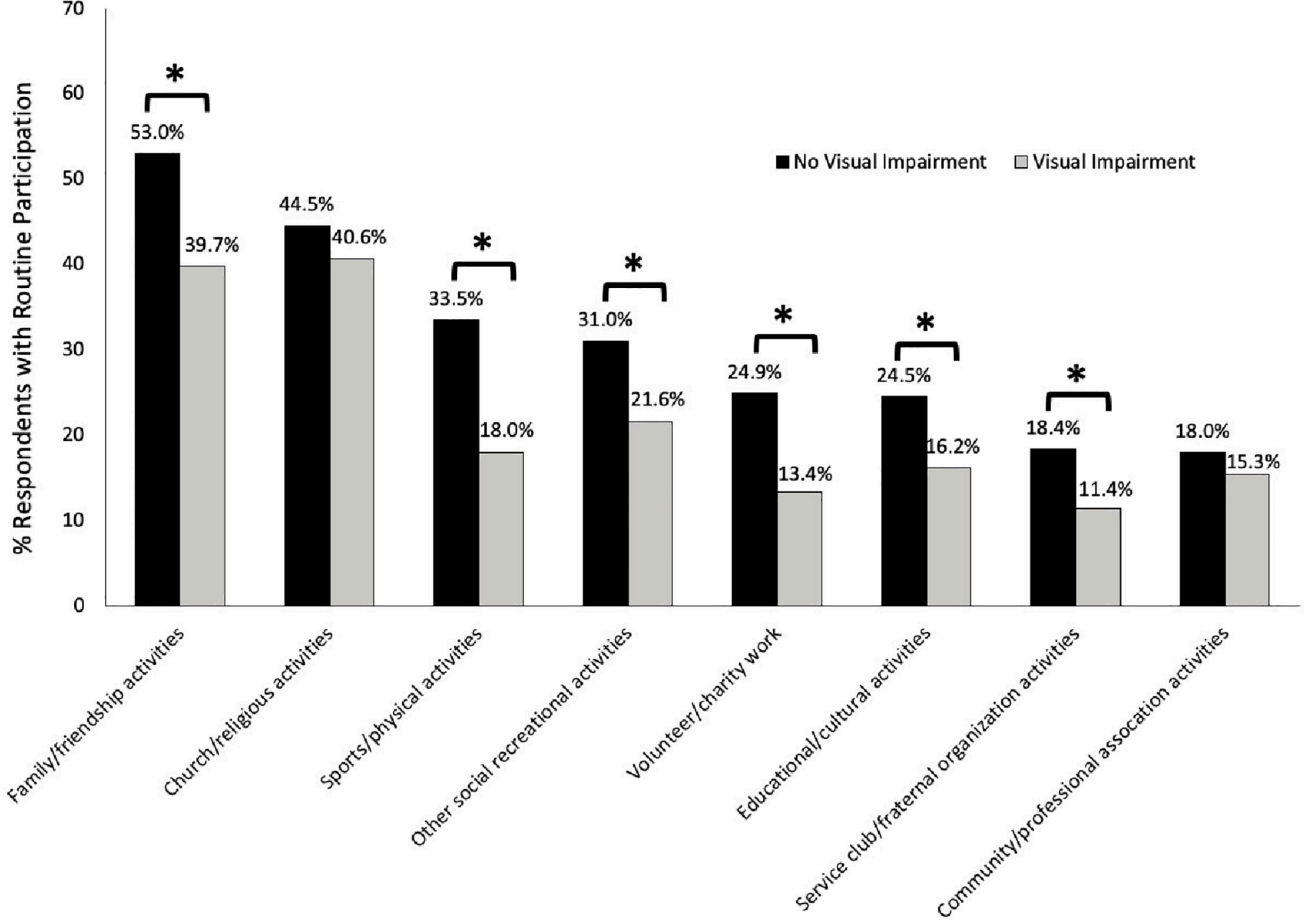
Participation in social activities for seniors 65 years or older with and without self-reported visual impairment. *p<0.05.

Seniors with self-reported VI experienced significantly reduced participation in six of eight activities surveyed: family/friendship activities, sports/physical activities, service club/fraternal organization activities, volunteer/charity work, educational/cultural activities, and participation in other social recreational activities compared to those without VI (p<0.05; Fig 1). When participation in family/friendship activities was re-defined as ‘at least once a day’, ‘at least once a week’, or ‘at least once a month’ as the other activities did, the difference in participation between seniors with (71.5%) and without self-reported VI (85.4%, p<0.05) was still significant. Differences in participation in church/religious activities and community/professional association activities were non-significant between seniors with and without self-reported VI (Fig 1). Overall, significantly more seniors with VI reported they did not participate in any of the eight activities surveyed compared to those without VI (10.1% vs 2.9%; p<0.05).

**Fig 2:**
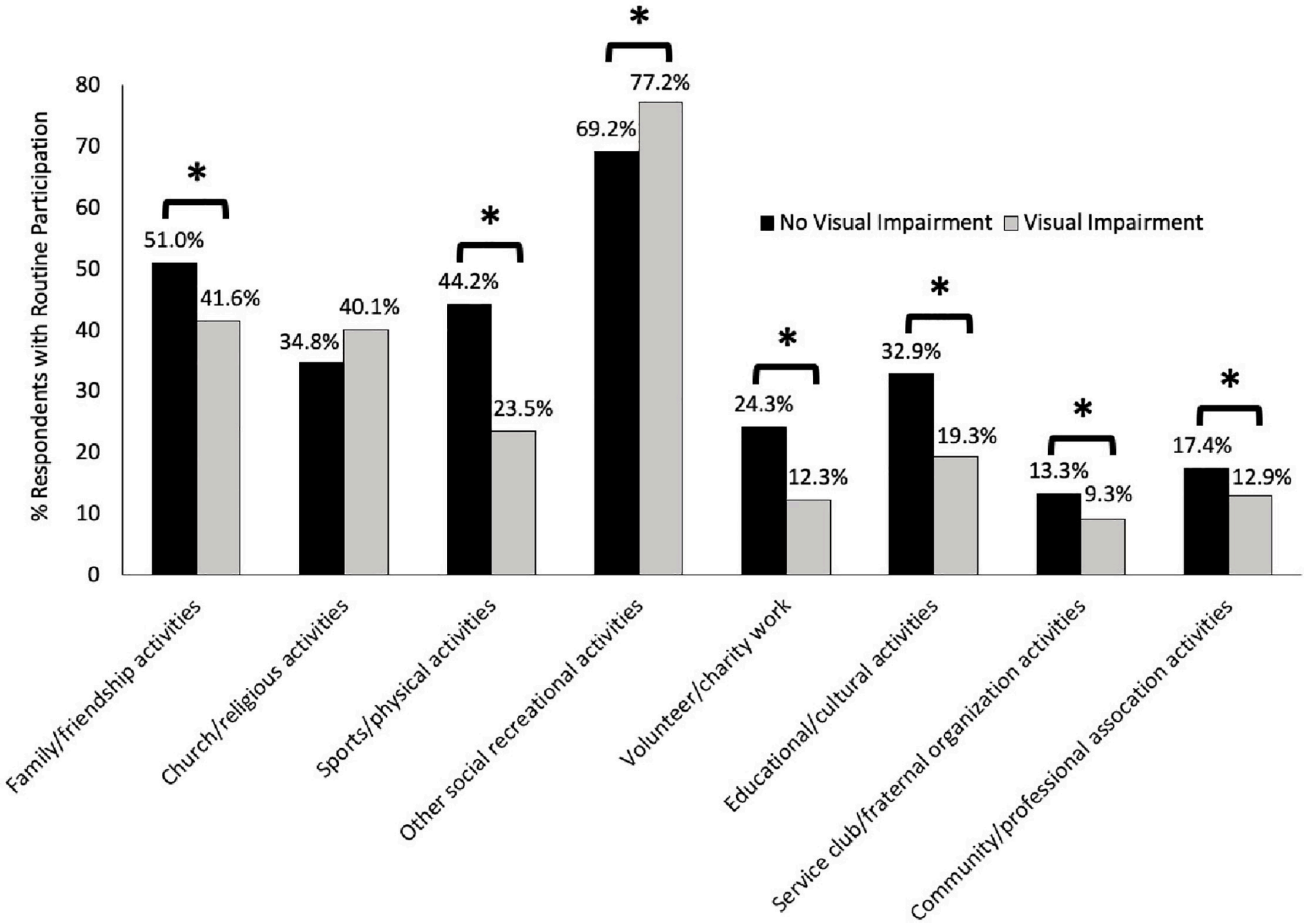
Participation in social activities for individuals 45 years or older with and without self-reported visual impairment. *p<0.05.

When the patterns of social participation were examined among people aged 45+, except for participation in church/religious activities, differences in all other activities between individuals with and without self-reported VI were statistically significant (Fig 2). No participation in any of the eight activities was not statistically significant for seniors over the age of 65 (3.9% vs 3.1% for people with and without glaucoma). However, no participation in any activity was statistically significant for individuals over the age of 45 (3.9% vs 1.8% for people with and without glaucoma; p<0.05)

### Social participation among people with and without glaucoma

**Fig 3:**
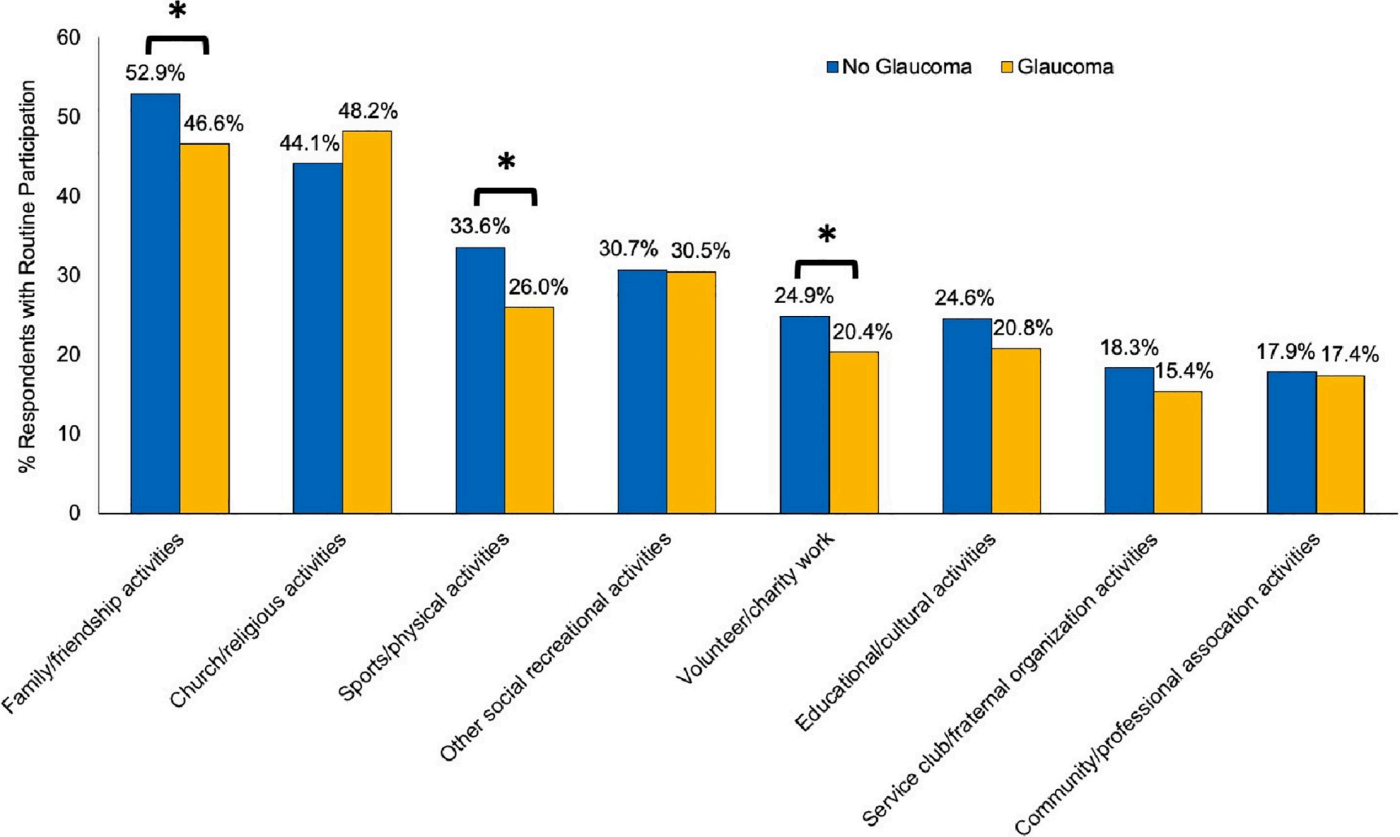
Participation in social activities for seniors 65 years or older with and without self-reported glaucoma. *p<0.05.

Seniors with glaucoma experienced significantly reduced participation in family/friendship activities, sports/physical activities and volunteer/charity work compared to those without glaucoma (p<0.05; Fig 3). When participation in family/friendship activities was re-defined as ‘at least once a day’, ‘at least once a week’, or ‘at least once a month’ similar the other activities, the difference between seniors with (83.0%) and without glaucoma (85.0%, p>0.05) was non-significant. Overall, the proportion of individuals who did not participation in any of the eight social activities was similar between seniors with and without glaucoma (3.9% vs 3.1%; p>0.05).

**Fig 4:**
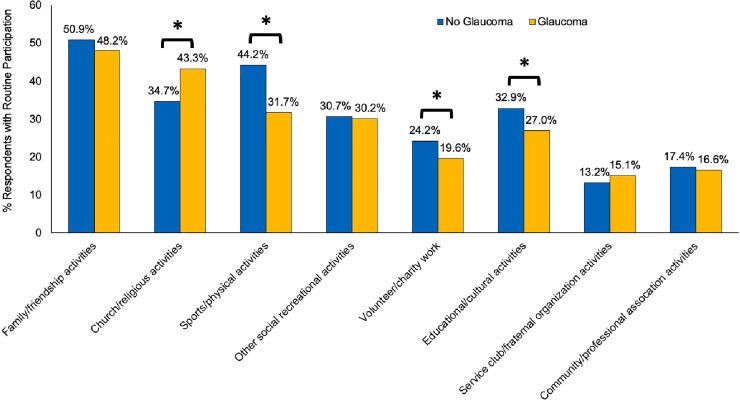
Participation in social activities for individual 45 years or older with and without self-reported glaucoma. *p<0.05.

The patterns among individuals aged 45+ (Fig 4) differed to some extent from the patterns among seniors. Compared to people without glaucoma, reduced participation in sports/physical activities and volunteer/charity work still remained among individuals with glaucoma, however, participation in family/friendship activities became similar (Fig 4) for glaucoma versus non-glaucoma. Furthermore, significantly different participations were noted in church/religious activities and educational/cultural activities. No participation in any of the eight activities became statistically significant (3.9% vs 1.8% for people with and without glaucoma, p<0.05).

### Multiple regression analyses

**Fig 5:**
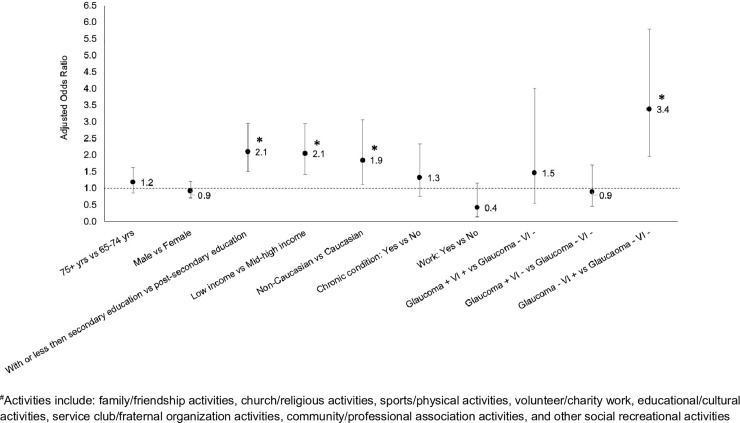
Adjusted odds ratio (aOR) of not participating in any of the social activities for seniors 65 years or older with different combinations of self-reported visual impairment (VI) and glaucoma. -: absence of the condition; +: presence of the condition. Vertical bar indicates the 95% confidence interval. Variables controlled for in the model include age, sex, highest level of education, total household income, ethnic background, current job status and chronic conditions other than self-reported VI and glaucoma.

When potential confounding effects of age, sex, highest level of education, total household income, ethnic background, current job status and chronic conditions other than self-reported VI and glaucoma were controlled for, seniors with self-reported VI and no glaucoma were more likely not to be involved in any social activities compared to seniors with no VI and no glaucoma (adjusted odds ratio (aOR) = 3.4, 95% CI 2.0–5.8; Fig 5). For seniors with glaucoma and no VI, their level of no participation in any of the eight social activities was similar to those with no VI and no glaucoma (p>0.05; Fig 5).

**Fig 6:**
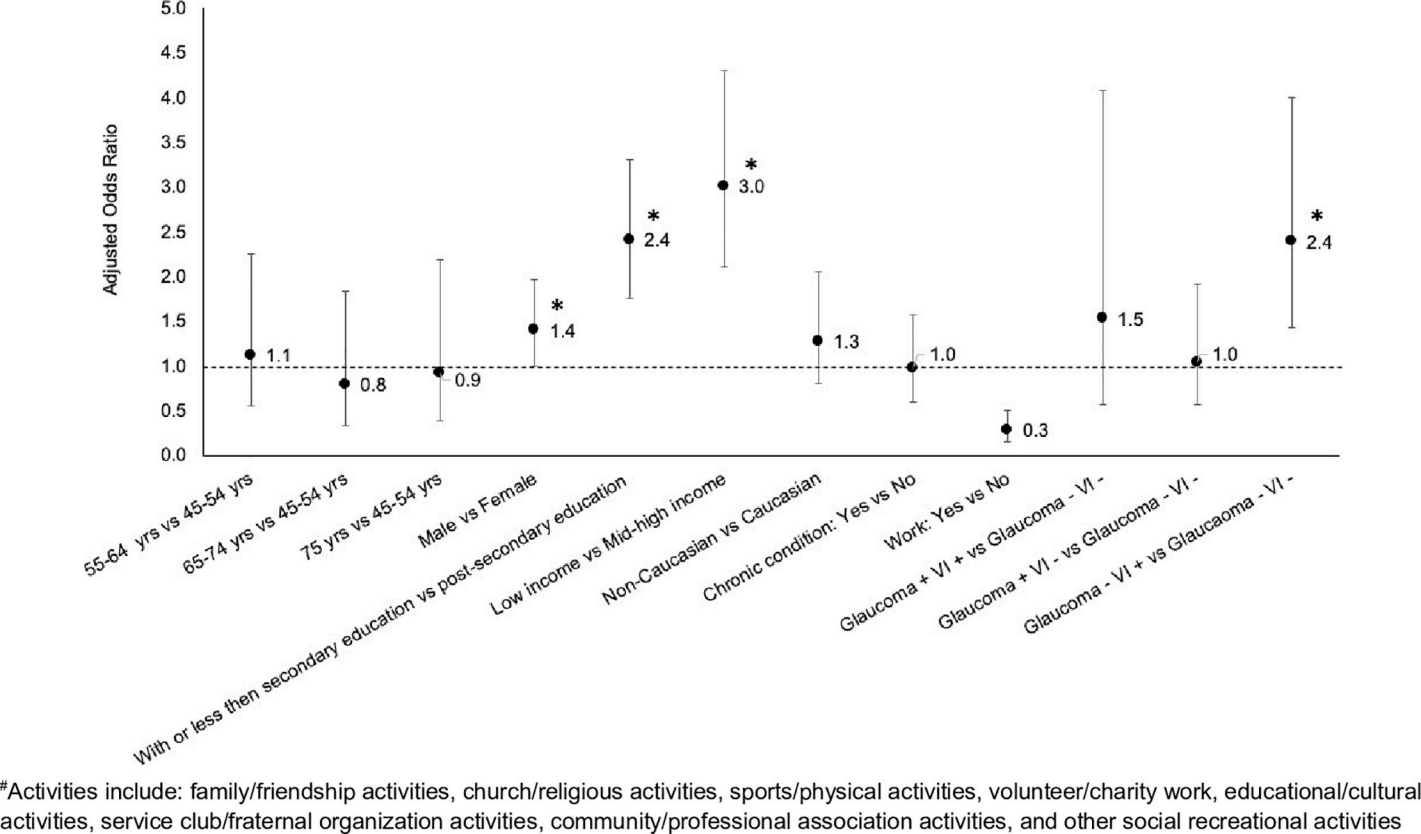
Adjusted odds ratio (aOR) of not participating in any of the social activities for individuals 45 years or older with different combinations of self-reported visual impairment (VI) and glaucoma. -: absence of the condition; +: presence of the condition. Vertical bar indicates the 95% confidence interval. Variables controlled for in the model include age, sex, highest level of education, total household income, ethnic background, current job status and chronic conditions other than self-reported VI and glaucoma.

Among individuals aged 45+, low levels of education and income and self-reported VI and no glaucoma were similarly associated with an increased risk of no participation in any of the eight social activities (Fig 6). However, the increased risk of no participation among non-Caucasians than Caucasians seen in people aged 65+ disappeared in people aged 45+.

## Discussion

This study examines the association between self-reported VI and glaucoma versus senior’s participation in a wide range of social and community activities. We report that seniors with self-reported VI had significantly reduced participation in sports/physical activities, family/friendship activities, service club/fraternal organization activities, volunteer/charity work and educational/cultural activities. Among seniors with self-reported glaucoma, we observed significantly reduced engagement with family/friends activities, sports/physical activities and volunteer/charity work. Seniors with self-reported VI and no glaucoma were more likely not to be involved in any social activities compared to those without self-reported VI and no glaucoma.

Our findings on reduced social participation in the elderly with self-reported VI are in line with previous reports.[35–38] In a recent study in Canada, Mick et al. assessed VI using the question “Is your eyesight, using glasses or corrective lenses if you use them, [excellent/very good/good/fair/poor or non-existent or blind]?” with answers of “fair” and “poor, non-existent or blind” as having vision loss.[35] They similarly found that vision loss among those aged 45 years or older was associated with no participation on a weekly basis in any of the 8 social activities we assessed, with an aOR of 1.2 compared to 2.4 from our study among individuals aged 45+ (Fig 6).[35] As suggested by Norton and associates, we consider the difference in the magnitude of aORs reported in the two studies are due to differences in assessment of VI, in definition of participation and in different co-variates included in the regression models.[44] We assessed VI using a series of questions while Mick et al’s study assessed VI using a single question only. We defined participation as involvement in an activity “at least once a day’, ‘at least once a week’, or ‘at least once a month’, while the study by Mick et al defined participation on a weekly basis. We reported differences in frequency and aOR of social participation in different activities and overall no participation between individuals with and without self-reported VI and those with and without glaucoma. The study by Mick et al only briefly mentioned the difference in aOR between people with and without self-reported VI. We focused on seniors who were significantly affected by VI and glaucoma and presented additional results those aged 45+. Mick et al’s study examined people aged 45+ only. As revealed by our results for those aged 65+ versus those aged 45+, differences exist between these two groups, suggesting that patterns in the 45+ group cannot be applied to the 65+ group.

In the US, Crews et al. studied seniors aged 70 years or older and reported that seniors with VI had significantly reduced OR for visiting friends in the past 2 weeks (0.7), phoning friends (0.8), attending church (0.7), going to movies (0.6), eating out (0.7), and exercising (0.7) compared to seniors with no vision and no hearing loss.[38] These findings are similar to ours except for church attendance where Crews et al’s study found reduced participation. We found seniors with and without VI experienced similar levels of participation in church/religious activities. Data in Crews’ study was collected in 1994 and our data was collected in 2008/2009. It is possible that the observed difference in church attendance may in part be explained by shifts in religious/spiritual behaviors in North America over the past 15 years, cultural differences between the US and Canada and the different age groups studied.

In the UK, Liljas studied VI among only senior men with VI, assessed with the question “using glasses or corrective lenses if needed, can you see well enough to recognize a friend at a distance of 12 feet/4 yards (across a road)?”.[36] This VI assessment differed from our assessment where a series of VI questions was asked and those with self-reported VI were defined as impairment uncorrected by lenses for distance vision, near vison or both, or no sight at all (see Methods). Liljas et al. reported that VI was associated with a higher rate of poor social interaction (age adjusted OR = 2.1), defined as participating in three or fewer of the nine activities they studied on a weekly basis.[36] In spite of the different definitions for VI and participation, the conclusions from the UK study are similar to ours.

We suggest potential reasons for seniors with VI having reduced participation may include reduced ability to ambulate (particularly in unfamiliar environments), fear of falling, transportation issues and psychological distress (i.e. depression, emotional distress and prolonged anxiousness).[45–51] The rate of self-reported depression in our study was significantly higher in seniors with self-reported VI (16.1%) versus those without self-reported VI (10.2%, p<0.05). The combination of mobility limitations, fears and distresses may lead VI seniors[52] to pursue safer sedentary activities and withdraw from participation in social activities.[53] It is also possible that VI may be a marker for other underlying health conditions (e.g. cardiovascular disease) or general biological aging. Therefore we cannot rule out the possibility that the observed reduced participation in seniors with self-reported VI may be attributed to vision-manifested other chronic health conditions.

Our study shows that compared to seniors without glaucoma, those with glaucoma experience significantly reduced participation in sports/physical activities, family/friendship activities and volunteer/charity work. This finding may be explained partly by reports that individuals with glaucoma walk more slowly, are more likely to bump into objects [30], have a greater fear of falling,[54] have a higher rate of falls,[29] have disruptions in their gaze-foot coordination[55], and are more home bound and are less likely to travel away from home.[56] Alone or in combination, these “activity limitations” may increase the chance that glaucoma patients choose not to participate in sports/physical activities, family/friendship activities outside the household and volunteer/charity work. The levels of social participation were similar between seniors with and without glaucoma across the other five activities surveyed. This suggests that glaucoma has less impact on social participation than VI.

There are limitations of this study. First, VI and glaucoma was self-reported, not clinically measured. Bias related to self-reporting cannot be ruled out. However, it is the individual’s self-reported vision (not their best corrected vision measured in clinics) that maintains an individual's day-to-day function. We feel self-reported VI may best reflect real-life situations. This concept is also in agreement with the revised definition for VI by WHO where the words ‘best corrected’ be replaced by “presenting”.[57,58] For glaucoma, MacLennan and colleagues compared the agreement between self-reported glaucoma and the glaucoma diagnosis documented in medical records among Americans aged 70 years and older.[59] The authors reported high agreement with a Cohen’s kappa of 0.73.[59] However, we do note that multiple vision measures contribute to a person’s level of self-reported vision.[60] Given the high prevalence of undetected glaucoma,[61] many elderly with undiagnosed glaucoma may have been misclassified as non-glaucoma by utilizing the self-report. This may be one potential reason for the non-significant findings in some areas of the activities examined. The second limitation is the cross-sectional design of the study makes it difficult to establish a causal link between self-reported VI (or glaucoma) and social participation. Thirdly, the survey did not ask why respondents chose not to participate in the activities; future research should investigate if non-participation is due to vision or non-vision related issues. Fourthly, besides glaucoma and cataracts, no other vision disorder questions were asked. As VI caused by cataracts may be included in our survey questions for VI (e.g., cannot read ordinary newsprint etc), cataracts were not studied separately. Lastly, the study data came from the past decade and may not reflect the most recent situation. However, it provides a historic picture to benefit for future comparisons and is the most recent data available in Canada.

A study strength is the nationwide and randomly selected sample. Common biases from clinic-based studies such as unrepresentative and/or smaller samples have been overcome. Furthermore, participation in social activities was investigated using a standardized set of eight questions.

In conclusion, the number of seniors with VI or glaucoma will likely increase substantially over the next twenty years.[39] We report that seniors with self-reported VI or glaucoma have significant reductions in participating in numerous social activities. In spite of reduced participation, individuals with glaucoma do value their engagement with diverse social activities,[62] demonstrating their desire to be involved in social activities. Enhancing social engagement is a modifiable factor for individuals, families, governments and societies. Results of this study thus provide a basis to promote the development of policies and programs that aim to increase the social involvement of seniors with VI and glaucoma, ultimately avoid social isolation and lead to successful aging in spite of having a vision disorder.
